# The contributing factors of tapered wedge stem alignment during mini-invasive total hip arthroplasty

**DOI:** 10.1186/s13018-015-0192-x

**Published:** 2015-04-21

**Authors:** Shinya Hayashi, Takaaki Fujishiro, Shingo Hashimoto, Noriyuki Kanzaki, Ryosuke Kuroda, Masahiro Kurosaka

**Affiliations:** Department of Orthopaedic Surgery, Kobe University Graduate School of Medicine, 7-5-1 Kusunoki-cho, Chuo-ku, Kobe 650-0017 Japan

**Keywords:** Tapered wedge stem, THA, Stem alignment, 3D template

## Abstract

**Background:**

Minimally invasive surgical approaches are widely used for total hip arthroplasty (THA). However, potential problems related to a reduced visual field during surgery, such as implant malposition, neurovascular injury, and poor implant fixation, have been reported. In these situations, a shorter stem is easier to insert in the femoral canal. To evaluate the accuracy of shorter stem orientation, we focused on the accuracy of stem orientation especially in short tapered wedge stems and evaluated the contribution factors of stem malalignment during mini-invasive total hip arthroplasty.

**Methods:**

One hundred ten hips that underwent THA with a Summit stem (58 hips) (DePuy, Warsaw, IN) as straight stem and TriLock stem (52 hips) (DePuy) as tapered wedge stem were enrolled in this study. For preoperative and postoperative evaluation, a CT scan of the pelvis and knee joint was obtained and was transferred to 3D template software. We compared the alignment of preoperative plan for stem anteversion/valgus/anterior tilt angles and postoperative stem alignment, and the absolute error was defined as the surgical error. To clarify the factors contributing to the malalignment or surgical error, we evaluated postoperative stem alignment and several associated factors.

Further, we compared the clinical parameters between two types of stems for analysis of the influence of stem type differences.

**Results:**

The mean absolute value of the alignment error (postoperative stem alignment-preoperative planning alignment) was not changed in the short tapered wedge and straight stems. Sex, age at operation, or original canal anteversion did not affect the accuracy of stem alignment. However, high body mass index (BMI) affected the accuracy of stem alignment. Clinical outcomes were not changed by the difference of stem types.

**Conclusion:**

The postoperative alignment of short tapered wedge stem was accurate, same as the straight stem during mini-invasive THA, but we need to pay attention when using this in obese patients.

## Background

Socket and stem orientation during total hip arthroplasty (THA) are critical factors for achieving an optimal range of motion and joint stability [1-3]. Although stem orientation can be estimated by the surgeon during the operation, the intraoperative estimation of stem alignment was found to have limited accuracy, even when an anatomically shaped cementless stem was used [4].

Classically, templating is performed using a plain anteroposterior (AP) radiograph of the pelvis [5]. However, because of the differences in individual patient anatomy and variations in magnification and projection, important radiographic parameters are not always reliable on plain radiographs. Sariali et al. reported on the high accuracy of hip anatomy restoration performed using a novel 3-dimensional (3D) CT scan-based technique for preoperative planning, and the results were comparable with those of navigation for stem alignment [6]. Even for the postoperative evaluation of implant orientation, the evaluation of stem orientation using CT data is very valuable. We recently showed that the accuracy of stem orientation based on CT-based fluoro-matched navigation can be assessed using postoperative CT data, and the clinical accuracy of CT-based fluoro-matched navigation is adequate for stem alignment orientation [7]. Thus, computer-assisted navigation is an effective tool to obtain precise information about stem orientation. However, the navigation system for THA is not popular worldwide because of its cost. Therefore, the factors contributing to stem alignment should be evaluated.

Minimally invasive surgical approaches are currently used for THA. Mini-incision THA reduced postoperative pain and blood loss, speed recovery, and reduce the hospital stay compared with THA using a standard approach [8]. However, some researchers are concerned that mini-incision THA may introduce new potential problems related to a reduced visual field during surgery, such as implant malposition, neurovascular injury, and poor implant fixation [9,10].

Actually, a shorter and thinner stem is easier to insert into the femoral canal when minimally invasive surgical approaches are used for THA. Therefore, surgical error should be used to evaluate the accuracy of intraoperative estimations especially in the case of shorter stems. In this study, we determined the absolute difference between the stem alignment estimated by surgeons intraoperatively and that measured using postoperative CT and evaluated the contribution factors of stem malalignment during total hip arthroplasty.

## Methods

### Patients and surgery

**Figure 1:**
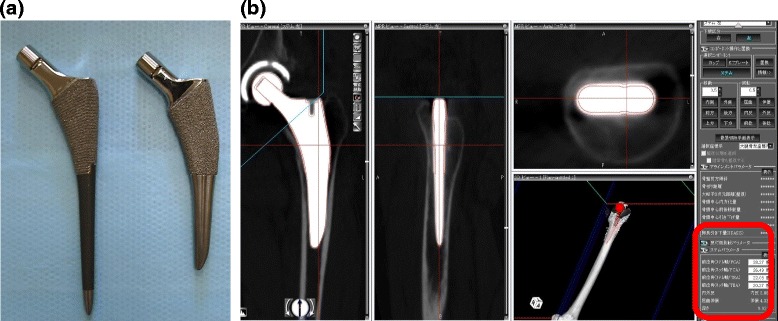
Materials. **(a)** Photographs of two different types of stem. Left: Summit stem (straight stem), right: TriLock stem (tapered wedge stem). **(b)** Postoperative CT data were transferred to the planning module and reconstructed in the axial, frontal, and sagittal planes. The computer-aided design model of the femoral implant was superimposed. The parameters of stem alignment are indicated by red circles.

**Figure 2:**
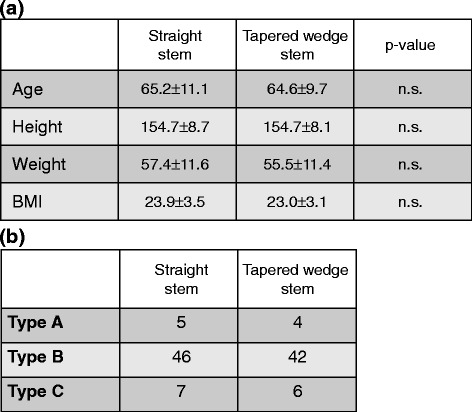
Patients’ background. **(a)** Demographic data of the study group. **(b)** Patient number according to Dorr classification for femur shape in straight and tapered wedge stem.

This study analyzed 110 hips in 21 men and 89 women. The patients underwent THA with a Summit stem (58 hips) (DePuy, Warsaw, IN; straight stem) between January 2011 and March 2012 and TriLock stem (52 hips) (DePuy; tapered wedge stem) between April 2012 and December 2013 for osteoarthritis (99 hips, developmental dislocation of the hip (DDH) 71 hips) or idiopathic osteonecrosis of the femoral head (11 hips) (Figure 1a). All surgeries were performed by the anterolateral approach with MIS (OCM approach). Briefly, skin incision was used over the anterior portion of the greater trochanter slightly curved (7 cm). Spread of anterolateral muscular interval without muscle resection, exposure of capsule, and capsular incision were performed. After neck osteotomy, acetabulum preparation and cup implantation were performed. For femoral preparation, leg position was in external rotation, hyperextension, and adduction. Capsular release nearby greater trochanter, stem implantation, repositioning, and wound closure were performed. The mean ages of patients at the time of surgery in the straight and tapered wedge stem groups were 65.2 ± 11.1 and 64.6 ± 9.7 years, respectively. The mean heights of patients in the straight and tapered wedge stem groups were 154.7 ± 8.7 and 154.7 ± 8.1 cm, respectively. The mean weights of patients in the straight and tapered wedge stem groups were 57.4 ± 11.6 and 55.5 ± 11.4 kg, respectively. The mean body mass index (BMIs) of patients in the straight and tapered wedge stem groups were 23.9 ± 3.5 and 23.0 ± 3.1 kg/m^2^, respectively. There were no significant differences in mean age, height, weight, and BMI between the straight and tapered wedge stem groups (Figure 2a). The proximal femoral shape may be critical for insertion of the stem. Therefore, we classified the cases according to Dorr femoral bone classification. The number of case in each type was similar between the straight and tapered wedge stem groups (Figure 2b).

### Preoperative planning and postoperative measurement of stem alignment

For preoperative and postoperative evaluations, a CT scan from the pelvis to the knee joint was performed and transferred to 3D template software (Zed hip, Lexi, Tokyo, Japan). Computer-aided design (CAD) models of the implants were manually adjusted for postoperative multi-planar reconstruction in CT images (Figure 1b). Stem anteversion, valgus, and anterior tilt angles were measured with respect to the mechanical axis of the femur. The mechanical axis was estimated from the center of both epicondyles of the femur and the femoral head. Preoperative planning of the stem alignment for original canal anteversion and zero degree of valgus/anterior tilt was performed preoperatively. Original canal anteversion was defined according to the methods of Sugano et al. [11]. Briefly, the femoral neck axis was calculated as the best-fit line connecting slices drawn through a central segment of the neck. Original canal anteversion was defined as the angle between the axis of the neck and a line connecting the epicondylar line. We compared the preoperative stem anteversion/valgus/anterior tilt angles and postoperative stem alignment, and the absolute error was defined as the surgical error.

### Postoperative measurement of clinical results

To analyze the influence of different types of stems on the clinical parameters after THA, we compared the clinical parameters (operation time, blood loss, complications, and Harris hip score at 1 month postoperatively) between the straight stem and tapered wedge stem. The parameter of complications included both intraoperative (fracture and crack) and postoperative (infection, dislocation, and symptmatic embolism) complications.

### Statistical analysis

All data are expressed as the mean ± standard deviation (SD) unless otherwise indicated. Statistical analysis was performed by using the Mann-Whitney *U* test for comparisons of paired samples. Correlations between the postoperative stem alignment error and original canal anteversion, age, height, weight, or BMI were examined by Pearson’s chi-square test. In all cases, p <0.05 was considered significant.

### Ethics

The study protocol was approved by the Kobe University Graduate School of Medicine Ethics Committee on 8 September 2011 (No 1220), and all patients provided informed consent.

## Results

### The postoperative stem alignment of short tapered wedge stem was accurate, same as the straight stem

**Figure 3:**
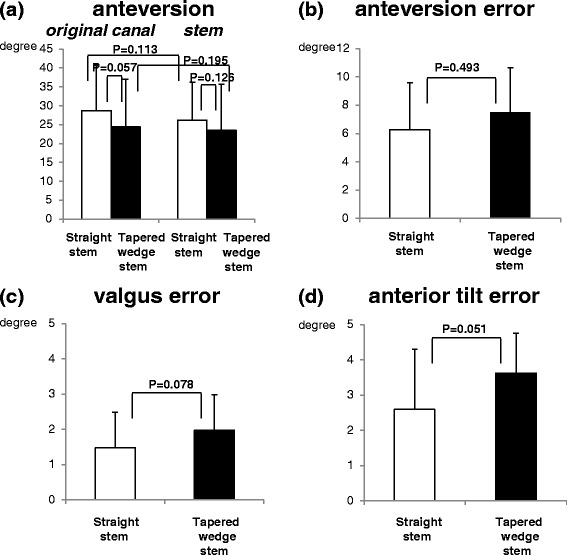
Accuracy of stem alignment. **(a)** Comparisons of the mean values of original canal and stem anteversions in the straight and tapered wedge stem groups. **(b-d)** Comparisons of absolute mean values of stem orientation errors in the straight and tapered wedge stem groups: **(b)** anteversion, **(c)** valgus, and **(d)** anterior tilt errors.

The mean original canal anteversion values were 28.7 ± 12.1° (straight stem) and 24.4 ± 12.6° (tapered wedge stem) (Figure 3a). The mean stem anteversion values were 26.2 ± 10.1° (straight stem) and 23.5 ± 12.2° (tapered wedge stem) (Figure 3a). There were no significant differences in the anteversion values between the straight and tapered wedge stems (Figure 3a). The mean absolute values of surgical error (postoperative stem anteversion-preoperative planning anteversion) were 6.3 ± 3.3° (straight stem) and 7.5 ± 3.1° (tapered wedge stem) (Figure 3b). The mean absolute values of valgus error were 1.5 ± 1.1° (straight stem) and 1.9 ± 1.5° (tapered wedge stem) (Figure 3c). The mean absolute values of anterior tilt error were 2.6 ± 1.7° (straight stem) and 3.6 ± 1.1° (tapered wedge stem) (Figure 3d). The mean absolute values of anteversion, valgus, or the anterior tilt error (as measured by postoperative stem alignment-preoperative planning alignment) were not significantly changed in the short tapered wedge and straight stems (Figure 3b-d).

### The accuracy of stem anterior tilt was affected by BMI

**Figure 4:**
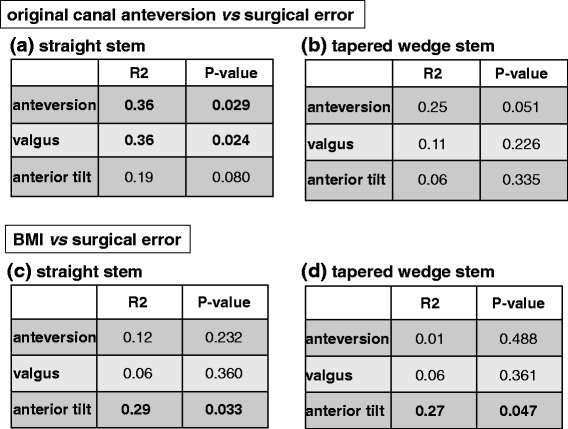
Correlation of stem alignment and various parameters. **(a, b)** Correlation between original canal anteversion and stem alignment with **(a)** straight stem and **(b)** tapered wedge stem. **(c, d)** Correlation between stem orientation errors and BMI with **(c)** straight stem and **(d)** tapered wedge stem.

To clarify the contributing factors for stem alignment during insertion, we measured the correlation between the surgical alignment error and original canal anteversion, age, height, and BMI. Surgical alignment errors were significantly correlated with the anteversion/valgus error and original canal anteversion in the straight stem, but not in the tapered wedge stem (Figure 4a, b). Age or height was not significantly correlated with surgical error (data not shown). However, there was significant correlation between the anterior tilt error and BMI in both types of stems (Figure 4c, d). These results indicate that obesity affects the accuracy of postoperative stem alignment.

### Clinical outcomes were not changed by the difference of stem types

**Figure 5:**
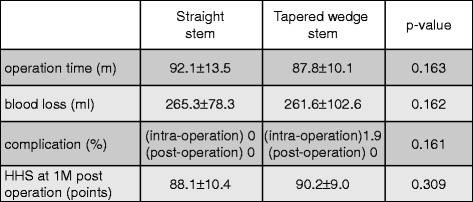
Demographic data of clinical outcomes.

The mean operation times were 92.1 ± 13.5° (straight stem) and 87.8 ± 10.1° (tapered wedge stem). The mean blood loss volumes were 265.3 ± 78.3° (straight stem) and 26.16 ± 102.6° (tapered wedge stem). The mean points of HHS at 1 month post operation were 88.1 ± 10.4° (straight stem) and 90.2 ± 9.0° (tapered wedge stem). There were no significant differences in the clinical results between the straight and tapered wedge stems (Figure 5). We also analyzed about the intraoperative complications (fracture and crack) and postoperative complications (infection, dislocation, and symptomatic embolism). There was no case of infection, dislocation, or symptomatic embolism. However, intraoperative calcar fracture was occurred in one case of tapered wedge stems (Figure 5).

## Discussion

The placement of the femoral stem in excessive anteversion or retroversion can result in a significant increase in the incidence of dislocation resulting from an impingement of the neck of the stem by the rim of the acetabular component [12]. Varus-valgus alignment is a critical factor in the use of cementless stems to avoid complications [13,14]. Vresilovic et al. demonstrated that varus alignment correlated with loosening of cementless stems [13], and Gill et al. demonstrated that varus alignment caused periprosthetic femoral insufficiency fractures [14]. Muller et al. showed that stem tilting in the sagittal plane has an influence on the position of the center of the femoral head and thus also on prosthesis anteversion [15]; therefore, the stem alignment of anteversion, varus-valgus, and sagittal tilt should be correctly considered to avoid complications.

Several studies assessing the accuracy of stem orientation during surgery using postoperative CT data have been published [4-16]. Dorr et al. demonstrated an underestimation of 1.5°. Wines and McNicol demonstrated that the difference between the estimated prosthetic and true anteversion was underestimated by 1.1°. Both studies showed that intraoperative estimations were generally accurate with a small difference of 1.0° and 1.5° between intraoperative and postoperative femoral anteversion. However, those series included cases with underestimation and overestimation. The accuracy of stem orientation should be evaluated using absolute values. Hirata et al. demonstrated that the mean absolute value of surgical error was 7.3° [17]. In this study, the mean absolute values of surgical error were 6.3° (straight stem) and 7.5° (tapered wedge stem), which are consistent with the values reported previously [17]. However, the average difference between preoperative planning and postoperative stem anteversion showed 2.5° (straight stem) and 0.9° (tapered wedge stem) underestimation. Hirata et al. demonstrated that the estimated prosthetic anteversion was significantly greater than the original canal anteversion by 5.8° [17]. The discrepancy could be attributed to the surgical approach. Wines et al. demonstrated that the stem anteversion was higher in the posterior approach than in the modified Hardinge approach [4]. The potential risk of posterior dislocation associated with the posterior approach could explain these observations [4]. Therefore, the average difference between the preoperative planning and postoperative stem anteversion was underestimated in both stems.

Schmidutz et al. demonstrated that only 58% of cases of modular short stem were placed in a neutral position (within 3°), whereas 98% of cases of normal length stem were placed in a neutral position [18]. The stem-shaft axis showed a wider range of varus-valgus positions in the short stem group than in the normal length stem group [18]. Kamada et al. reported a similar tendency in the Mayo short stem [19]. In our study, we demonstrated that the mean absolute error was unchanged between the short tapered wedge and straight stems. The width of the frontal plane is designed wider in the short tapered wedge stem than in other types of short stems such as the Mayo short stem. Therefore, the alignment of the short tapered wedge stem was well controlled.

The femoral anteversion of Asians is generally larger than that of Caucasian [20,21]. Dorr et al. reported that the primary indications for THA were primary osteoarthritis in 98 hips (89.9%) and DDH in only 5 hips (4.6%) in their study [16]. In the present study, a higher number of DDH hips (64.5%) were included, and the mean stem anteversion values were 28.7° ± 12.1° (straight stem) and 24.4° ± 12.6° (tapered wedge stem). We demonstrated a significant correlation between the anteversion/valgus error and original canal anteversion in the straight stem, but not in the tapered wedge stem. The length of the straight stem is relatively long, and then, the alignment of stem may be affected by femoral canal shape such as excessive anteversion, whereas the alignment of short tapered stem can be controlled easily by the surgeon during insertion.

Elson et al. demonstrated that the morbidly obesity in patients undergoing THA increases the risk of varus-valgus femoral stem malpositioning [22]. Operating on a patient with a high BMI makes it difficult to identify bony landmarks because of the excess adipose tissue. In the present study, the accuracy of stem anterior tilt during insertion was affected by BMI in the straight and tapered wedge stems. Therefore, BMI might affect stem alignment, although BMI and stem design do not compromise the stem alignment.

Watts et al. demonstrated that the insertion of uniquely exaggerated proximal taper angle stem (ProxiLock) had an increased risk of postoperative periprosthetic femur fracture compared with non-ProxiLock stem group [23]. We also analyzed the influence of different types of stems on the clinical parameters after THA. However, we did not find any differences of clinical outcomes including postoperative periprosthetic femur fracture.

## Conclusion

The alignment of the short tapered wedge stem was well controlled. However, high BMI affected the alignment of short tapered wedge stem. We therefore need to pay attention to obese patients when using the short tapered wedge stem during mini-incision THA.
